# Bioactive Equivalence of Combinatorial Components Identified in Screening of an Herbal Medicine

**DOI:** 10.1007/s11095-013-1283-1

**Published:** 2014-02-19

**Authors:** Peng Liu, Hua Yang, Fang Long, Hai-Ping Hao, Xiaojun Xu, Ying Liu, Xiao-Wei Shi, Dan-Dan Zhang, Hao-Chuan Zheng, Qian-Ying Wen, Wen-Wen Li, Hui Ji, Xi-Juan Jiang, Bo-Li Zhang, Lian-Wen Qi, Ping Li

**Affiliations:** 1 State Key Laboratory of Natural Medicines, China Pharmaceutical University, Nanjing, 210009 China; 2 Tianjin University of Traditional Chinese Medicine, Tianjin, 300193 China

**Keywords:** bioactive equivalence, Cardiotonic Pill, combinatorial components screening, herbal medicines

## Abstract

**Purpose:**

To identify bioactive equivalent combinatorial components (BECCs) in herbal medicines. The exact composition of effective components in herbal medicines is often elusive due to the lack of adequate screening methodology. Herein, we propose a hypothesis that BECCs accounting for the whole efficacy of original herbal medicines could be discovered from a complex mixture of constituents.

**Methods:**

We developed a bioactive equivalence oriented feedback screening method and applied it to discover the BECCs from an herbal preparation Cardiotonic Pill (CP). The operations include chemical profiling of CP, followed by an iterative loop of determining, collecting and evaluating candidate BECCs.

**Results:**

A combination of 18 compounds was identified as BECCs from CP, which accounts for 15.0% (w/w) of original CP. We have demonstrated that the BECCs were as effective as CP in cell models and in a rat model of myocardial infarction.

**Conclusions:**

This work answers the key question of which are real bioactive components for CP that have been used in clinic for many years, and provides a promising approach for discovering BECCs from herbal medicines. More importantly, the BECCs could be extended to improve quality control of herbal products and inspire an herbal medicines based discovery of combinatorial therapeutics.

**Electronic supplementary material:**

The online version of this article (doi:10.1007/s11095-013-1283-1) contains supplementary material, which is available to authorized users.

## Introduction

Herbal medicines have played an important role in health maintenance and disease treatment for thousands of years (1,2). Accompanying with hot discussions on developing multidrug therapy for multi-gene diseases, herbal medicines are receiving increasing attention worldwide because they have long been postulated as multicomponent therapeutics in clinical practice (3–7). Nevertheless, the exact composition of effective components is often elusive due to the lack of adequate screening methodology. A variety of research efforts in recent decades have been focused on isolating and identifying single effective constituents from herbal medicines. However, they disregarded the combinatorial role and integrative therapeutic effects of multiple active compounds (8,9). Generally, the therapeutic efficacy of herbal medicines is achieved by combinatorial components rather than single compound. Hence, it is incumbent upon researchers to elucidate the exact combinatorial composition of effective components in herbal medicines, if any, accounting for of the holistic efficacy of herbal medicines.

In this work, we propose a hypothesis that bioactive equivalent combinatorial components (BECCs) accounting for the whole efficacy of original herbal medicines could be discovered from a complex mixture of constituents. To explore BECCs from herbal medicines, we have developed and described herein a bioactive equivalence oriented feedback screening method, and applied it to discover the BECCs from an herbal preparation Cardiotonic Pill (CP, also known as the Compound Danshen Dripping Pill). CP is a Chinese herbal medicinal preparation in which Salviae Miltiorrhizae Radix and other two Chinese medicines, namely Notoginseng Radix and borneolum, were combined in a fixed ratio using modern techniques of pharmaceutical preparation. CP has been widely applied clinically in China to improve cardiac function and coronary circulation for the therapy of angina pectoris (10,11) and has recently been approved to enter Phase III clinical trials by the FDA (ClinicalTrials.gov Identifier: NCT01659580). Herein, we have identified a combination of 18 compounds as BECCs out of 36 identified components and other unidentified minor components, which were as effective as CP in cell models and in a rat model of myocardial infarction (MI). The content of BECCs accounts for 15.0% (w/w) of original CP, and could be considered as the “defined labeled amount” of active component combination for CP.

This work offers evidence-based data to answer the key question of which are real bioactive components for Cardiotonic Pill that have been used in clinic for many years. While the nature of this study refers specifically to Cardiotonic Pill, we suggest that this screening method is promising for detecting BECCs for other herbal medicines, and more importantly, to improve quality control of herbal medicines and inspire an herbal medicines based discovery of combinatorial therapeutics.

## Methods

### Chemical Profiling of CP

To select candidate BECCs from CP, we initially profiled the chemical constituents of CP. Three batches of CP (Tasly Pharmaceutical Co., Ltd., Lot: 100824, 110419, 110510) were obtained from local drug stores (Nanjing, China). Reference compounds were obtained from the National Institute for The Control of Pharmaceutical and Biological Products (Beijing, China) or previously preserved in our laboratory. The purity of each reference compound was determined to be higher than 98%. For HPLC-UV/MS analysis, CP (15 mg, without addition of excipients) was extracted using 75% (v/v) ethanol (3 ml) for 30 min by ultrasonication and subsequently centrifuged at 13,000 rpm for 10 min. The supernatant was analyzed on an Agilent 1100 series HPLC system (Agilent, USA). The separation was performed on an Agilent Zorbax SB-C18 column (250 × 4.6 mm, 5 μm) using the 0.02% formic acid in water (A) and 0.02% formic acid in ACN (B). The gradient elution program was 10–22% B at 0–10 min, 22–23% B at 10–18 min, 23–24% B at 18–23 min, 24–27% B at 23–33 min, 27–33% B at 33–40 min, 33–41% B at 40–52 min, 41–70% B at 52–54 min, 70–72% B at 54–62 min, 72–100% B at 62–70 min, 100% B at 70–77 min. The flow rate was kept at 0.5 ml/min at 30°C. For segmental monitoring (12) based on UV, different detection wavelengths were performed for different periods of time: 203 nm for 0–57 min; 281 nm for 57–77 min. MS spectra were acquired on a 6530 Q-TOF mass spectrometer (Agilent, USA) equipped with an electrospray ionization (ESI) interface. The operating parameters were as follows: drying gas (N_2_) flow rate, 10.0 L/min; drying gas temperature, 320°C; nebulizer, 45 psig; capillary, 3,500 V; skimmer, 65 V; OCT RFV, 750 V; and fragmentor voltage 120 V. For MS/MS experiments, the collision energy was adjusted from 15 to 70 V to optimize signals and obtain maximal structural information from the ions of interest. The sample was analyzed in both positive and negative modes. The mass range was set at m/z 100–3,000.

### Trapping and Preparing Candidate BECCs

To obtain the combination of candidate BECCs, we applied the real-time components trapping and combining system for sample preparation (Figure S1, Supplementary Material). A preparative Agilent 1100 series HPLC system was coupled to a LEAP Shell fraction collection system (LEAP Technologies, USA). The fraction collection system was composed of an autosampler (HTC PAL MXY 04-01A), a system control software (LEAP Shell 3) and a fraction collector (HTC PAL MXY 013-02A). The HPLC separation was performed on an Agilent Zorbax SB-C18 semi-preparative column (250 × 9.4 mm, 5 μm) with a flow rate of 2 ml/min, and other HPLC conditions were the same as those in chemical profiling of CP.

After the injection of 20 μl CP at the concentration of 100 mg/ml in 75% (v/v) ethanol, candidate BECCs were prepared using the real-time components trapping and combining system (Figure S1, Supplementary Material) according to the collection program as following: P2 at 648–690 s, P3 at 996–1,050 s, P5 at 1,308–1,380 s, P6 at 1,386–1,446 s, P7 at 1,548–1,620 s, P8–P9 at 1,866–1,992 s, P10 at 2,001–2,052 s, S3 at 2,058–2,130 s, P11 at 2,256–2,382 s, P12 at 2,640–2,772 s, S5 at 3,060–3,102 s, S9 at 3,312–3,348 s, S12 at 3,402–3,438 s, T3 at 4,110–4,146 s, T4–T5 at 4,356–4,440 s, T7 at 4,560–4,602 s. Candidate BECCs were collected at position II while the remaining part was collected at position I. Subsequently, the solvent was removed using a speedvac evaporator (Genevac EZ-2 plus, Genevac Technologies, UK). The samples were reconstituted at a concentration as in original CP for method validation and bioactivity assay.

### Bioactivity Assays *In Vitro*

To assess the bioactive equivalence, we evaluated the bioactivities of candidate BECCs and the original CP simultaneously. Three cell models related to angina pectoris were used (13,14), including oxidized low density lipoprotein (Ox-LDL)-mediated human umbilical vein endothelial cell (HUVEC) injury (15), lipopolysaccharide (LPS)-stimulated RAW 264.7 macrophage inflammation (16), and simulated ischemia (SI)-induced H9c2 cardiomyocytes injury (17). If the 90% confidence interval (CI) of relative efficacy compared to original CP fell within the range of 70–143% (18), the candidate BECCs were considered to be a bioactive equivalent with original CP.

HUVEC (EA.hy926), mouse macrophage (RAW 264.7) and rat cardiomyoblast (H9c2) cell lines were purchased from American Type Culture Collection (ATCC, USA). Cells were grown at 37°C under a humidified atmosphere with 5% (v/v) CO_2_ in accordance to optimal media and growth conditions specified by ATCC.

To assess the effect of candidate BECCs on HUVECs, we pretreated cells with various samples (corresponding concentrations in 0.4 mg/ml Cardiotonic Pill) or Trolox (100 μM, as a positive control) for 12 h. After preincubation, supernatant was removed and cells were subjected to Ox-LDL (70 μg/ml, XieSheng Biotechnology, China) for 24 h except the vehicle control group. Cell viability was determined by cell counting kit-8 (CCK-8, Dojindo Molecular Technologies Inc., Japan). Supernatant was collected for lactate dehydrogenase (LDH) assay (Jiancheng Bioengineering Institute, China) according to the manufacturer’s instructions. ROS production in HUVECs was determined by a fluorometric assay using DCFH-DA (Beyotime, China) as previously reported (19).

To assess the anti-inflammatory effect of candidate BECCs, the macrophages were pretreated for 2 h with various samples (corresponding concentrations in 0.5 mg/ml Cardiotonic Pill) or luteolin (20 μM, as a positive control) and then stimulated for 20 h with LPS (1 μg/ml). The NO production was determined as nitrite concentration in the culture medium according to a Griess reaction (20). The accumulated interleukin-6 (IL-6) and prostaglandin E2 (PGE2) in the culture medium were measured using commercial ELISA kits (R&D, USA) according to the manufacturer’s instructions.

To evaluate the protective effect of candidate BECCs on cardiomyocytes against simulated ischemia injury, a rat H9c2 cardiomyoblasts simulated ischemia model was established (17). After overnight incubation, the cells were pretreated for 24 h with various samples (corresponding concentrations in 0.1 mg/ml Cardiotonic Pill). To mimic the *in vivo* conditions of myocardial ischemia, the cells were incubated in a buffer containing 116.4 mM NaCl, 5.4 mM KCl, 1.8 mM CaCl_2_, 0.8 mM MgSO_4_, 2.6 mM NaH_2_PO_4_, 26.2 mM NaHCO_3_, 20.l mM HEPES and then exposed to 2% O_2_−93% N_2_−5% CO_2_ for 6 h. After the treatment, cell viability was determined by CCK-8 assay (Dojindo Molecular Technologies Inc., Japan), according to the manufacturer’s instructions. Cells cultured in complete medium under a normoxic atmosphere served as a control. The treatment concentrations were chosen based on the optimal concentration of Cardiotonic Pill (Figure S2, Supplementary Material).

### Bioactivity Assays *In Vivo*

To further investigate whether the candidate BECCs (III) are a bioactive equivalent with original formulation in terms of *in vivo* efficacy, we utilized a rat model of MI (21–24). All animal procedures were approved by the Animal Ethics Committee of China Pharmaceutical University. Male Sprague-Dawley rats (250–300 g) were purchased from the Aier Maite Technology Corporation (Suzhou, China). The rats were housed in humidity- and temperature- controlled environment with a 12 h light: 12 h dark cycle. Water and standard laboratory diet were available *ad libitum*.

MI was produced by the ligation of the left anterior descending (LAD) coronary artery as reported previously (21,22). Rats were randomly assigned into seven groups: four treatment groups (CP, candidate BECCs (III), mixture of reference compounds (MRCs) (III), remaining part (III)), a MI group, and a sham group. Briefly, various samples (corresponding concentrations in 20 mg/kg Cardiotonic Pill) in the treatment group were given once daily by oral administration for 3 days before MI. The sham and MI group received only saline. For MI operation, rats were anesthetized with chloral hydrate (300 mg/kg i.p.) and ventilated by a respirator (HX-100E, Taimeng Co. Ltd., China) with a tidal volume of 10 ml/kg and a respiratory rate of 80 cycles per minute. A left thoracotomy was performed in the fourth intercostal space, and then MI was induced by ligation of the LAD artery 2 mm from the tip of the left auricle. The sham-operated rats underwent the same thoracotomy without LAD ligation. Electrocardiogram was recorded before and after operation procedures. Coronary occlusion was confirmed by ST segment elevation in the electrocardiogram and the appearance of epicardial cyanosis.

To evaluate the bioactivity of candidate BECCs (III) on cardiac function, left ventricular (LV) catheterization was performed (25). At the end of 6 h ischemic period, a catheter was inserted into the left ventricle for evaluating cardiac function. Left ventricular end diastolic pressure (LVEDP), left ventricular systolic pressure (LVSP), +*dP*/*dt* and -*dP*/*dt* were recorded through a biological mechanic experiment system (BL420, Taimeng Co. Ltd., China). Cardiac marker enzymes including creatine kinase-MB (CK-MB) and LDH were tested for estimation of myocardial cell damage using commercial kits (Jiancheng Bioengineering Institute, China) (26). To investigate the bioactivity of candidate BECCs (III) on inflammatory response, tumor necrosis factor-α (TNF-α) level in serum was measured using a commercial ELISA kit (R&D, USA) (27). At the end of experiment, the heart was excised, sliced into five sections, and infarct size was measured using 2, 3, 5-triphenyltetrazolium chloride (TTC) staining to assess myocardial injury as reported previously (28,29). Sections were photographed and the area at risk was quantified with the aid of the Image-Pro Plus 6 software.

### Metabolomic Profiling

To validate the assessment results of bioactive equivalence between BECCs and original CP, metabolomics strategy was applied to profile the metabolites in rat myocardial tissues using gas chromatography/time-of-flight mass spectrometry (GC/TOF MS) coupled to partial least square-discriminant analysis (PLS-DA). Rat hearts (*n* = 4–6) were immediately snap-frozen in liquid nitrogen and stored at −80°C until metabolomic profiling. The same extraction, derivatization, and analysis procedures were applied as described previously (30,31). Myocardial tissue (20 mg) from infart area was homogenized in a tube containing 800 μl methanol. [1, 2-^13^C_2_] Myristic acid was added to each tube as the internal standard. With an Agilent 7683 autosampler, the derivatized samples were analyzed using an Agilent 6890N GC system (Agilent, USA) coupled with a Pegasus III mass spectrometer (Leco, USA). The injector temperature was set at 270°C and helium was used as carrier gas at a constant flow rate of 1 ml/min. Column temperature was initially maintained at 70°C for 2 min and then was increased at a rate of 35°C/min to 305°C, where it was held for 2 min. The transfer line temperature was set at 250°C and ion source temperature at 200°C for the mass spectrometer. A 70-eV electron beam at a current of 2.0 mA were used for ions generation. The GC/TOF MS data of metabolites profiling of myocardial tissue were analyzed by PLS-DA, using the SIMCA-P 11.0 (Umetrics, Sweden). For PLS-DA modeling, samples were divided into different groups (e.g., sham, MI, BECCs and CP) as the qualitative “dummy” variable, Y.

### Statistical Analysis

All bioactive data were presented as mean ± standard deviation (SD). Data were subjected to statistical analysis using Graphpad Prism 6.0. One-way analysis of variance (ANOVA) with Dunnett’s *post*-*hoc* test was carried out for statistical comparison. In all cases, a *P*-value < 0.05 was considered to be significant.

For bioactive equivalence assessment, all the bioassay results were transformed to efficacy value according to the following equation:

$$ Efficac{y}_{drug}=\frac{\mathrm{Abs}\left( Activit{y}_{drug}- Activit{y}_{model}\right)}{\mathrm{Abs}\left( Activit{y}_{control}- Activit{y}_{model}\right)}\times 100\% $$

where *Activity*_*drug*_, *Activity*_*model*_ and *Activity*_*control*_ were the measured values of drug group (candidate BECCs, MRCs or original CP treated), model group (Ox-LDL-mediated HUVEC injury, LPS-stimulated RAW 264.7 macrophage inflammation, simulated ischemia-induced H9c2 cardiomyocytes injury or myocardial infarction) and control group respectively in a given bioassay. Bioactive equivalence was evaluated by calculating 90% confidence interval (CI) of the ratio between the efficacies of candidate BECCs and CP (two one-sided *t* test, see more information in Supplementary Material). If the 90% CI of relative efficacy compared to original CP fell within the range of 70–143% (18), the candidate BECCs were considered to be a bioactive equivalent with original CP.

## Results

### Chemical Profiling of CP

**Fig. 1:**
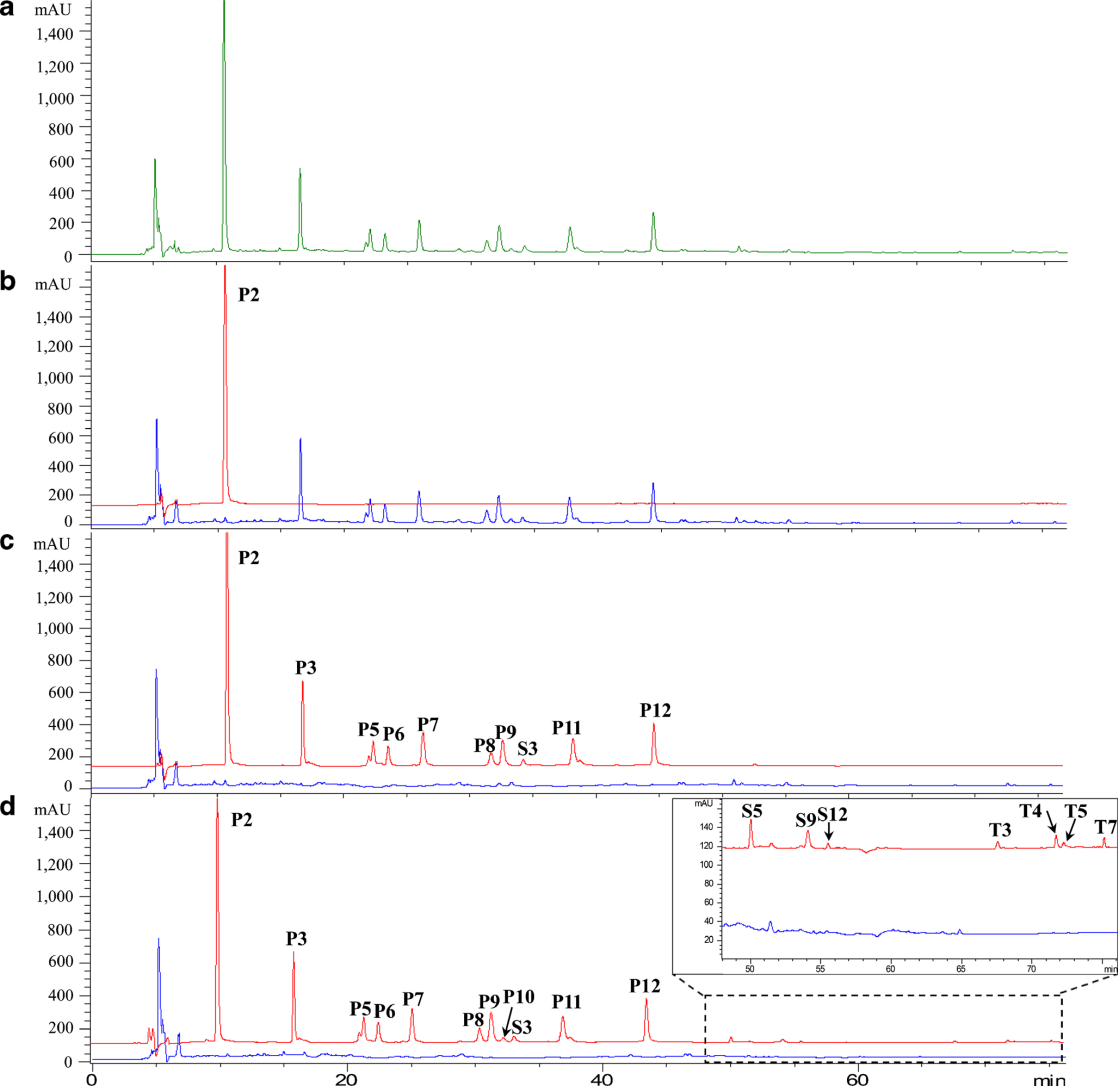
Validation of candidate BECCs in the three rounds of screening by HPLC-UV. Samples of Cardiotonic Pill (depicted in *green*), candidate BECCs (*red*) and the remaining part (*blue*) were analyzed at the same concentration. UV wavelengths: 203 nm for 0–57 min; 281 nm for 57–77 min. The peaks in the chromatograms are assigned the same numbers as in Figure S3, Supplementary Material. (**a**) Chromatogram of Cardiotonic Pill. (**b**) Chromatogram of candidate BECCs (I) and remaining part (I). Candidate BECCs (I) consisted of one compound, identified as tanshinol. (**c**) Chromatogram of candidate BECCs (II) and remaining part (II). Candidate BECCs (II) consisted of 10 compounds (mainly phenolic acids). (**d**) Chromatogram of candidate BECCs (III) and remaining part (III). Candidate BECCs (III) consisted of 18 compounds, including 10 phenolic acids, 4 saponins and 4 tanshinones. *BECCs* bioactive equivalent combinatorial components.

A variety of components were detected at HPLC-UV chromatogram using the segmental monitoring method (Fig. 1a). Structural characterization was performed by Q-TOF MS, and the total ion chromatograms of CP in positive and negative ion modes were shown in Figure S3, Supplementary Material. In total, 36 compounds in CP were detected and identified by comparison with available reference compounds or literature information, including 13 phenolic acids, 15 saponins and 8 tanshinones (Table I) (32,33).

**Table I:** The Retention Time, MS Data and Characterization of Identified Phenolic Acids, Saponins and Tanshinones in Cardiotonic Pill

Peak no.	t_R_ (min)	[M-H]^−^(m/z)	Theoretical (m/z)	Error (ppm)	Formula	Identification
P1	8.85	197.0450	197.0455	−2.77	C_9_H_10_O_5_	(*s*)-3-(3,4-dihydroxyphenyl) lactic acid
P2^a^	9.76	197.0453	197.0455	−1.25	C_9_H_10_O_5_	Tanshinol
P3^a^	15.38	137.0246	137.0244	1.33	C_7_H_6_O_3_	Protocatechuic aldehyde
P4^a^	16.60	179.0351	179.0350	0.66	C_9_H_8_O_4_	Caffeic acid
P5^a^	20.70	537.1032	537.1038	1.12	C_27_H_22_O_12_	Isolithospermic acid A
P6^a^	21.89	537.1041	537.1038	−0.34	C_27_H_22_O_12_	Isolithospermic acid B
P7^a^	24.73	417.0830	417.0827	0.67	C_20_H_18_O_10_	Salvianolic acid D
P8^a^	29.40	339.0518	339.0510	2.28	C_18_H_12_O_7_	Salvianolic acid G
P9^a^	30.30	359.0780	359.0772	2.11	C_18_H_16_O_8_	Rosmarinic acid
P10^a^	30.67	537.1045	537.1038	1.58	C_27_H_22_O_12_	Lithospermic acid
S1^a^	31.04	977.5309	977.5327	1.62	C_47_H_80_O_18_	Notoginsenoside-R1
S2^a^	33.82	991.5497	991.5483	−1.47	C_48_H_82_O_18_	Ginsenoside-Re
S3^a^	34.15	845.4912	845.4904	−1.09	C_42_H_72_O_14_	Ginsenoside-Rg1
P11^a^	34.91	717.1461	717.1465	−0.55	C_36_H_30_O_16_	Salvianolic acid B
P12^a^	41.63	493.1146	493.1140	−1.21	C_26_H_22_O_10_	Salvianolic acid A
P13^a^	46.67	491.0990	491.0984	1.28	C_26_H_20_O_10_	Salvianolic acid C
S4^a^	50.10	845.4899	845.4904	−0.01	C_42_H_72_O_14_	Ginsenoside-Rf
S5^a^	52.13	1,153.6005	1,153.6011	1.04	C_54_H_92_O_23_	Ginsenoside-Rb1
S6^a^	52.76	815.4792	815.4798	1.12	C_41_H_70_O_13_	Notoginsenoside-R2
S7^a^	53.63	829.4951	829.4955	0.48	C_42_H_72_O_13_	Ginsenoside-Rg2
S8	54.43	1,123.5878	1,123.5906	2.61	C_53_H_90_O_22_	Ginsenoside-Rb3
S9^a^	55.15	683.4379	683.4376	−0.56	C_36_H_62_O_9_	Ginsenoside-Rh1
S10^a^	55.56	1,123.5907	1,123.5906	−1.31	C_53_H_90_O_22_	Ginsenoside-Rb2
S11^a^	56.07	683.4370	683.4376	0.98	C_36_H_62_O_9_	Ginsenoside-F1
S12^a^	57.21	991.5507	991.5483	−2.39	C_48_H_82_O_18_	Ginsenoside-Rd
S13^a^	59.76	829.4959	829.4955	−0.20	C_42_H_72_O_13_	Ginsenoside-F2
S14^a^	60.45	829.4963	829.4955	−0.88	C_42_H_72_O_13_	20(S)-Ginsenoside Rg3
S15^a^	60.78	829.4936	829.4955	2.18	C_42_H_72_O_13_	20(R)-Ginsenoside Rg3
T1^a^	62.10	311.1288	311.1278	2.84	C_19_H_18_O_4_	Tanshinone IIB
T2	63.61	337.1429	337.1434	1.70	C_21_H_20_O_4_	Danshenxinkun D
T3^a^	66.81	279.1021	279.1016	1.86	C_18_H_14_O_3_	15,16-Dihydrotanshinone I
T4^a^	71.01	277.0864	277.0859	2.96	C_18_H_12_O_3_	Tanshinone I
T5^a^	71.55	297.1496	297.1485	2.75	C_19_H_20_O_3_	Cryptotanshinone
T6^a^	72.92	279.1023	279.1016	−2.56	C_18_H_14_O_3_	Methylenetanshinquinone
T7^a^	74.48	295.1334	295.1329	1.77	C_19_H_18_O_3_	Tanshinone IIA
T8^a^	75.01	283.1704	283.1693	−4.07	C_19_H_22_O_2_	Miltirone

*P1*–*P13* phenolic acids; *S1*–*S15* saponins; *T1*–*T8* tanshinones. t_R_: retention time

^a^Compared with reference compounds

### Determination of Candidate BECCs from CP

In our experiment, normalized peak area ratio (%) was chosen as the selection criterion. According to the HPLC chromatogram of CP, the selection threshold was set at 10% of the total peak area in the first round and only one peak, identified as tanshinol, was trapped and prepared, labeled as candidate BECCs (I) (Fig. 1b). When the selection threshold was reduced to 1% or 0.1%, 10 or 18 peaks were prepared as candidate BECCs (II) or candidate BECCs (III), respectively (Fig. 1c and d). Candidate BECCs (II) mainly consisted of phenolic acids, while candidate BECCs (III) consisted of 10 phenolic acids, 4 saponins and 4 tanshinones. Remaining part (III) of CP without candidate BECCs (III) mainly consisted of highly polar compounds such as carbohydrates, and other minor constituents.

### Trapping and Preparing Candidate BECCs

Three batches of candidate BECCs based on the described selection threshold were prepared through the corresponding collection programs. Using a semi-preparative column, the system could provide samples at the milligram level per HPLC run, which was adequate for cell tests. For our experiments in rats, a 1 week preparation period was required to accumulate sufficient samples using this system. The prepared samples were dried and reconstituted with 75% (v/v) ethanol to the original concentration for HPLC validation. As shown in Fig. 1, candidate BECCs were trapped and prepared from CP completely.

The preparation process may potentially lead to sample loss because of possible adsorption to the chromatographic column and compound degradation during heating processes. Thus, we investigated the preparative recovery and repeatability for each compound in the candidate BECCs (Table S1, Supplementary Material). The preparative recovery ranged from 87.7% to 97.9%, while the average recovery rate was approximately 92.9%. The preparative process showed stable repeatability with a RSD < 6.6%. This result indicates that the described system is feasible and reliable.

### *In Vitro* Assessment of Bioactive Equivalence Between Candidate BECCs and Original CP

**Fig. 2:**
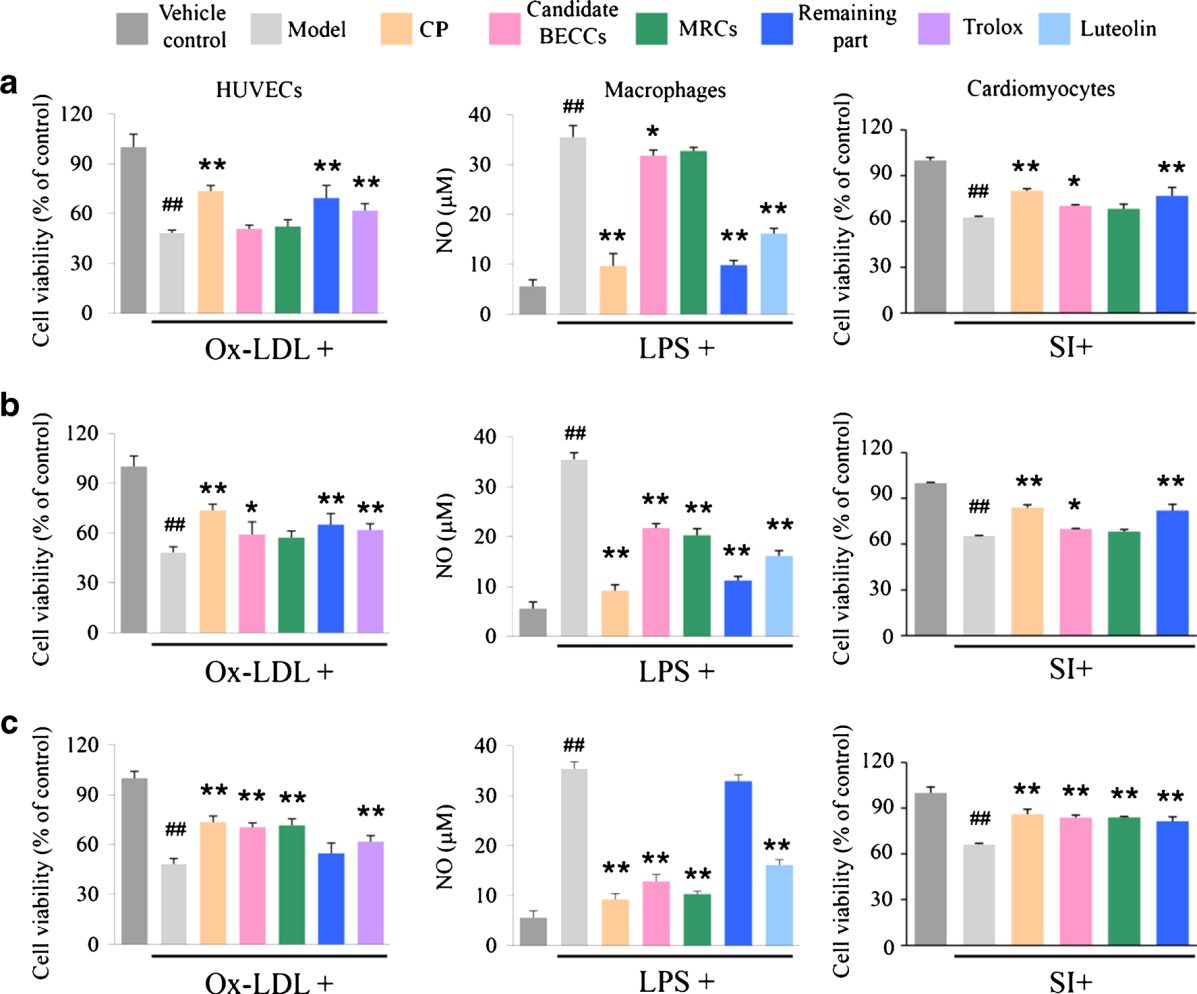
*In vitro* activity assays of candidate BECCs in the three rounds of screening. Samples in (**a**) the first round of screening, (**b**) the second round of screening, (**c**) the third round of screening were investigated in three cell models. HUVEC injury was induced by incubation with Ox-LDL and cell viability (as % of control) was assayed by CCK-8. Effects of samples on NO production of LPS-activated murine macrophage RAW264.7 cells was determined by Griess reaction. After 6 h of simulated ischemia, cell viability of H9c2 cardiomyoblasts was assayed by CCK-8. Trolox and luteolin were used as a positive control in HUVECs and macrophages respectively. Results are expressed as mean ± SD of at least three independent experiments. *P < 0.05, **P < 0.01 versus model group. ## *P* < 0.01 versus vehicle control group (one-way ANOVA, Dunnett test). Cells in model group were untreated. *CP* Cardiotonic Pill; *BECCs* bioactive equivalent combinatorial components; *MRCs* mixture of reference compounds; Remaining part: CP lacking candidate BECCs; *Ox*-*LDL* oxidized low density lipoprotein; *LPS* lipopolysaccharide; *SI* simulated ischemia.

CP showed remarkable protective effects in the three cell models, while candidate BECCs (I) exerted weak effects (Fig. 2a) and could not achieve bioactive equivalence as compared to the original CP (Table II). As such, we adjusted our feedback loop by reducing the selection threshold to 1% of total peak area and performed the second round of screening. Candidate BECCs (II) comprised of 10 compounds showed some activities (Fig. 2b). However, in the bioactive equivalence assessment, candidate BECCs (II) failed to meet the 70–143% requirements (Table II). For example, we observed a notable anti-inflammatory effect of candidate BECCs (II) in macrophages compared with model group (Fig. 2b, *P* < 0.0001, *n* = 3, one-way ANOVA, Dunnett test), but 90% CI of efficacy in suppressing the production of nitric oxide (NO) was 49.0–56.0%, and demonstrated that candidate BECCs (II) could not achieve bioactive equivalence with CP. In the third round of screening, the selection threshold was further reduced to 0.1% of total peak area, candidate BECCs (III) of 18 compounds increased the cell viability of damaged HUVECs and cardiomyocytes, and also alleviated the LPS-induced increase of NO in macrophages compared with model group (Fig. 2c, in HUVECs, *P* < 0.0001; in macrophages, *P* < 0.0001; in cardiomyocytes, *P* < 0.0001, *n* = 3, one-way ANOVA, Dunnett test). In the bioactive equivalence assessment, 90% CI for the efficacies of candidate BECCs (III) in all the cell tests fell within 70–143%, as listed in Table II. At the same time, the low activity of remaining part (III) also suggested that most of the bioactive components of CP were included in candidate BECCs (III).

**Table II:** Bioactive Equivalence Evaluation of Candidate BECCs and MRCs by *In Vitro* Assays

*In vitro* assay	Candidate BECCs (I)	MRCs (I)	Candidate BECCs (II)	MRCs (II)	Candidate BECCs (III)	MRCs (III)
HUVECs						
Cell viability	2.1–13.2%	1.5–23.6%	20.7–58.8%	23.7–44.8%	77.0–100.4%	77.8–107.7%
LDH	–	–	–	–	79.6–93.6%	73.5–91.1%
ROS	–	–	–	–	78.6–85.1%	73.7–79.0%
Macrophages						
NO	10.5–18.0%	8.4–13.0%	49.0–56.0%	53.1–63.1%	80.6–92.3%	91.8–100.4%
IL-6	–	–	–	–	81.7–84.1%	78.6–81.0%
PGE2	–	–	–	–	120.1–150.0%	147.1–185.3%
Cardiomyocytes						
Cell viability	38.3–47.8%	13.7–52.1%	22.5–27.3%	8.2–23.0%	75.6–105.9%	77.3–104.8%

*n* = 3. Bioactive equivalence was evaluated by two one-sided *t* test. The 90% CI of relative efficacy of candidate BECCs and MRCs compared to original CP was showd in the table. If the 90% CI of relative efficacy fell within the range of 70–143%, the candidate BECCs were considered to be a bioactive equivalent with original Cardiotonic Pill

*BECCs* bioactive equivalent combinatorial components; *MRCs* mixture of reference compounds

**Fig. 3:**
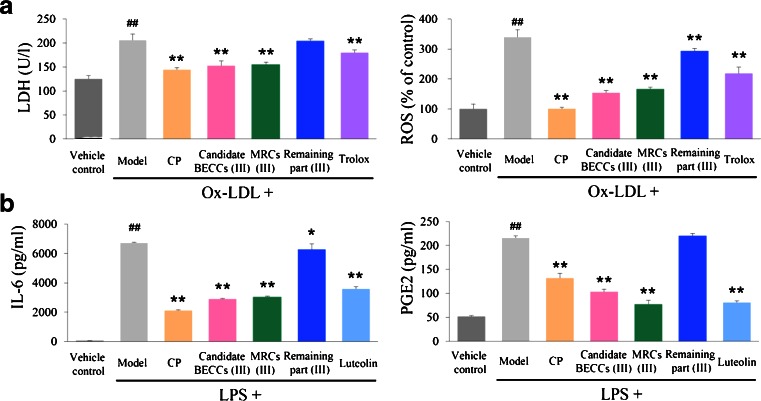
Effects of Candidate BECCs (III) on LDH and ROS in HUVECs and IL-6 and PGE2 in macrophages. (**a**) HUVECs were incubated with vehicle, CP, candidate BECCs (III), MRCs (III), remaining part (III) and trolox for 12 h, following 24-h stimulation of Ox-LDL. Supernatant of HUVECs was collected for LDH assay by a commercial kit. ROS production (as % of increase) in HUVECs was determined by a fluorometric assay using DCFH-DA as a probe. (**b**) Macrophages were pretreated with vehicle, CP, candidate BECCs (III), MRCs (III), remaining part (III) and luteolin for 2 h, following 20-h stimulation of LPS. The accumulated IL-6 and PGE2 in the culture medium were measured using commercial ELISA kits. Trolox and luteolin were used as a positive control in HUVECs and macrophages respectively. Results are expressed as mean ± SD of at least three independent experiments. *P < 0.05, **P < 0.01 versus model group. ## *P* < 0.01 versus vehicle control group (one-way ANOVA, Dunnett test). Cells in model group were untreated. *CP* Cardiotonic Pill; *BECCs* bioactive equivalent combinatorial components; *MRCs* mixture of reference compounds; Remaining part: CP lacking candidate BECCs; *Ox*-*LDL* oxidized low density lipoprotein; *LPS* lipopolysaccharide.

Ox-LDL increased LDH release and reactive oxygen species (ROS) concentration in HUVECs substantially, whereas preincubation with CP or candidate BECCs (III) notably attenuated the Ox-LDL-induced damage (Fig. 3a, CP versus Ox-LDL group, *P* < 0.0001 for LDH assay, *P* < 0.0001 for ROS assay; candidate BECCs (III) versus Ox-LDL group, *P* < 0.0001 for LDH assay, *P* < 0.0001 for ROS assay, *n* = 3, one-way ANOVA, Dunnett test). Additionally, results were consistent with the cell viability assay (Fig. 2c). To investigate the biological activities on macrophages, LPS stimulation resulted in a substantial increase of IL-6 and PGE2 secretion, while pre-incubation of CP or candidate BECCs (III) alleviated the LPS-induced increase of these inflammatory factors (Fig. 3b, CP versus LPS group, *P* < 0.0001 for IL-6 assay, *P* < 0.0001 for PGE2 assay; candidate BECCs (III) versus LPS group, *P* < 0.0001 for IL-6 assay, *P* < 0.0001 for PGE2 assay, *n* = 3, one-way ANOVA, Dunnett test). The results suggested that candidate BECCs (III) had a similar efficacy to CP.

In the bioactive equivalence assessment, 90% CI showed that ratios of efficacies between CP and candidate BECCs (III) in most cases lay within the acceptance range of 70-143% (Table II). Thus, candidate BECCs (III) could be considered as BECCs of CP *in vitro*.

To exclude the contribution of any undetected peaks masked in the candidate BECCs collected, we prepared a mixture of reference compounds (MRCs) with the identical compositions of candidate BECCs (Table S2, Supplementary Material) for bioactivity validation. Similar with candidate BECCs (III), MRCs (III) showed marked protective effects in all three cell models (Figs. 2c and 3). The bioactive equivalence assessment data were also consistent with that of candidate BECCs (III); 90% CI of efficacies in most cases for MRCs (III) lay within 70–143% (Table II).

**Fig. 4:**
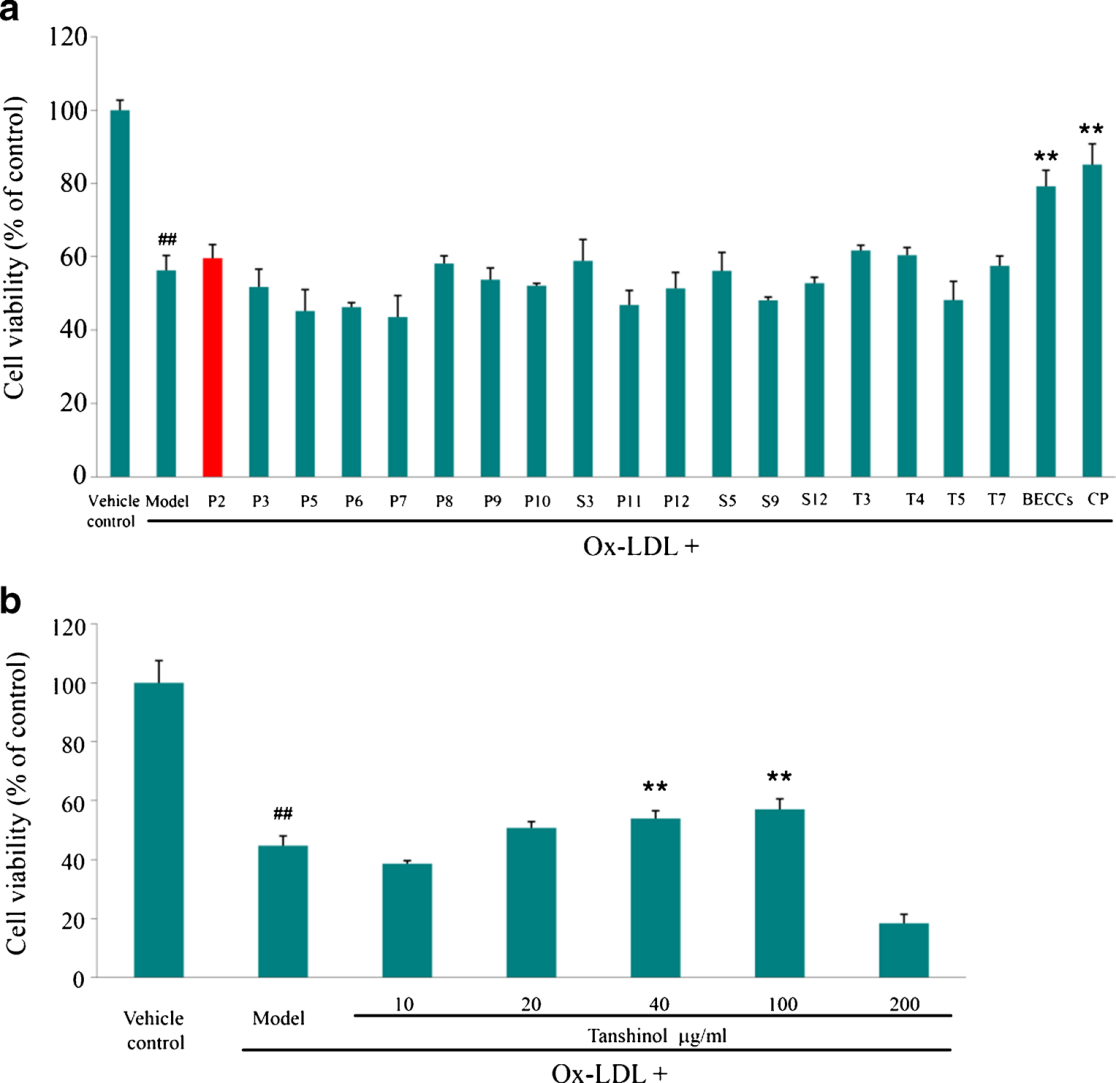
Protective effects of compounds in BECCs on HUVECs against Ox-LDL injury. HUVEC injury was induced by pretreatment with Ox-LDL for 24 h and cell viability (as % of control) was assayed by CCK-8. (**a**) Protective effects of 18 individual compounds. The concentrations of 18 compounds were equal to their concentrations in 0.4 mg/ml Cardiotonic Pill. Compound P2 is tanshinol with a concentration of 10.7 μg/ml (marked in *red*). (**b**) Protective effects of tanshinol on HUVECs at different doses. Results are expressed as mean ± SD of at least three independent experiments. **P < 0.01 versus Ox-LDL group. ## *P* < 0.01 versus vehicle control group (one-way ANOVA, Dunnett test). Cells in model group were untreated. *BECCs* bioactive equivalent combinatorial components; *CP* Cardiotonic Pill; *Ox*-*LDL* oxidized low density lipoprotein.

When the 18 compounds in candidate BECCs (III) were given individually at the concentration as in the original CP, none of these compounds showed substantial protective effects on HUVECs (Fig. 4a). For example, tanshinol, one of the most abundant compounds in CP, demonstrated weak protective effects on HUVECs at the same dose level as in the formulation (10.7 μg/ml). When the dose increased to 40 μg/ml, tanshinol showed some activity on HUVECs (Fig. 4b, tanshinol versus Ox-LDL group, *P* = 0.0027, *n* = 3, one-way ANOVA, Dunnett test), but it was not able to achieve bioactive equivalence with CP, even up to 100 μg/ml. Candidate BECCs (III) consisted of three groups of ingredients, including 10 phenolic acids, 4 saponins and 4 tanshinones. In order to test whether each group of constituents is necessary, phenolic acids, saponins or tanshinones were removed from candidate BECCs (III) respectively, and the combination of any two groups could not show equivalent bioactivity to original CP (Figure S4, Supplementary Material). The results indicated that each group of ingredients was indispensable.

### *In Vivo* Assessment of Bioactive Equivalence Between Candidate BECCs and Original CP

**Fig. 5:**
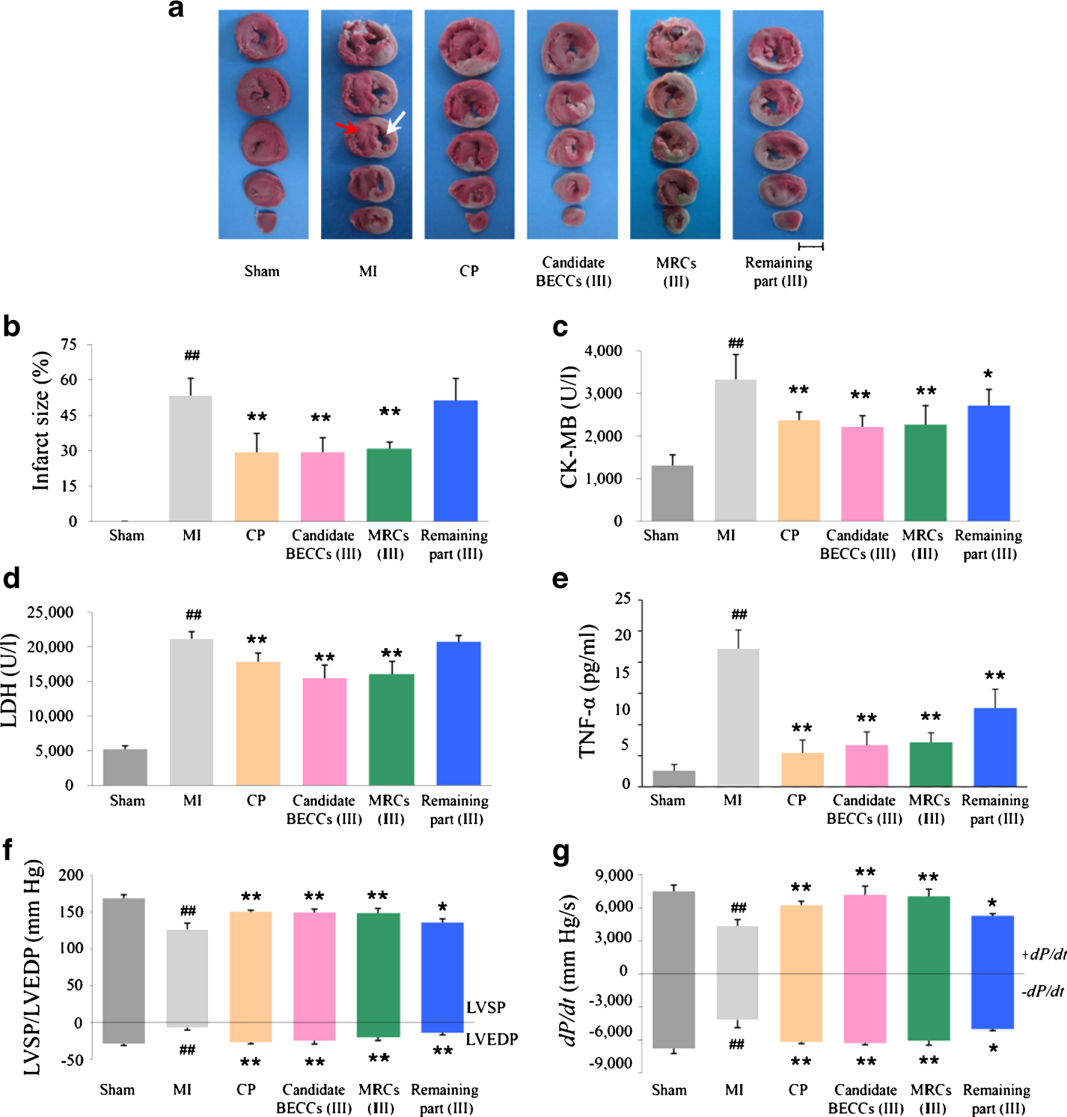
Candidate BECCs (III) showed comparable cardio-protective effects with CP against myocardial infarction. (**a**) Representative pictures of 2, 3, 5-triphenyltetrazolium chloride-stained myocardial sections (scale bar, 0.5 cm). *Red arrow* indicates non-infarct region while *white arrow* indicates infarct region. *n* = 5. (**b**) Quantification of infarct size of different samples. *n* = 5. (**c**) CK-MB, (**d**) LDH and (**e**) TNF-α levels of different samples were determined by commercial kits. *n* = 8. Left ventricular function including (**f**) LVEDP, LVSP and (**g**) + *dP*/*dt*, -*dP*/*dt*. Results are expressed as mean ± SD, *n* = 8. *P < 0.05, **P < 0.01 versus MI group. ## *P* < 0.01 versus sham group (one-way ANOVA, Dunnett test). *Sham* Sham operated; *MI* myocardial infarction; *CP* Cardiotonic Pill; *BECCs* bioactive equivalent combinatorial components; *MRCs* mixture of reference compounds; Remaining part: CP lacking candidate BECCs.

TTC-stained hearts were shown in Fig. 5a. Pretreatment with CP significantly reduced the infarct size compared with the MI group (Fig. 5b, 29.4 ± 4.0% versus 53.3 ± 3.7%, *P* = 0.0016, *n* = 5, one-way ANOVA, Dunnett test). Candidate BECCs (III) showed a similar effect of decreasing the infarct size to 29.4 ± 3.0% (Fig. 5b, *P* = 0.0016, *n* = 5, one-way ANOVA, Dunnett test). MI resulted in marked elevation in the serum levels of CK-MB and LDH (Fig. 5c and d). CP and candidate BECCs (III) inhibited the MI-induced increases in the activities of these enzymes (Fig. 5b, CP versus MI group, *P* = 0.0004 for CK-MB assay, *P* = 0.0017 for LDH assay; candidate BECCs (III) versus MI group, *P* < 0.0001 for CK-MB assay, *P* < 0.0001 for LDH assay, *n* = 8, one-way ANOVA, Dunnett test). These results indicate that the cardio-protective effect of candidate BECCs (III) is comparable to that of CP *in vivo*.

MI resulted in a substantial increase of TNF-α, while pre-incubation of CP or candidate BECCs (III) attenuated the elevation of TNF-α level (Fig. 5e, CP versus MI group, *P* < 0.0001; candidate BECCs (III) versus MI group, *P* < 0.0001, *n* = 8, one-way ANOVA, Dunnett test). Compared with the sham group, LVEDP was increased while LVSP, maximal rate of increase of LV pressure (+*dP*/*dt*) and maximal rate of decrease of LV pressure (-*dP*/*dt*) were decreased post-MI (Fig. 5f and g). Treatment with candidate BECCs (III) exhibited significant improvements on the heart’s diastolic and systolic functions (Fig. 5f and g).

According to the bioactive equivalence assessment, candidate BECCs (III) suggested comparable efficacy with original CP within 90% CI (Table III). The low activity of remaining part (III) also indicated that most of the bioactive components of CP were included in candidate BECCs (III) (Fig. 5). Furthermore, MRCs (III) containing the same constituents as candidate BECCs (III) (Table S2, Supplementary Material) showed marked protective effects against MI (Fig. 5). Bioactive equivalence data were also consistent with that of candidate BECCs (III) (Table III).

**Table III:** Bioactive Equivalence Evaluation of Candidate BECCs (III) and MRCs (III) in a Rat Model of Myocardial Infarction

*In vivo* assay	Candidate BECCs (III)	MRCs (III)
Infarct size	86.1–115.9%	80.2–109.2%
CK-MB	131.8–153.0%	104.2–135.9%
LDH	117.2–248.8%	110.5–188.4%
LVSP	84.8–107.8%	76.2–100.0%
LVEDP	82.6–97.2%	73.4–87.8%
+*dP*/*dt*	141.3–166.6%	127.2–156.5%
−*dP*/*dt*	98.3–111.6%	84.9–109.4%
TNF-α	84.5–100.3%	81.5–99.2%

*n* = 5–8. Bioactive equivalence was evaluated by two one-sided *t* test. The 90% CI of relative efficacy of candidate BECCs (III) and MRCs (III) compared to original CP was showd in the table. If the 90% CI of relative efficacy fell within the range of 70–143%, the candidate BECCs were considered to be a bioactive equivalent with original Cardiotonic Pill

*BECCs* bioactive equivalent combinatorial components; *MRCs* mixture of reference compounds

The results of metabolomic profiling were given as PLS-DA score plot that represents the metabolomics similarities of the samples (Fig. S5). The score plot of PLS-DA showed that both BECCs- and CP-treated groups were close to the sham group. It was demonstrated that the therapeutic efficacy of candidate BECCs (III) against MI is comparable to that of original CP.

## Discussion

Natural products and herbal medicines have played an important role in human health care and treatment of diseases for thousands of years (1,2). Herbal medicines contain complex and relatively unrefined mixtures of compounds, the holistic efficacy of herbal medicines is usually a product of the unresolved integrative effects between the constituents (3–7). It is not surprising that the previous efforts in isolating and screening single compounds from herbal medicines are less satisfactory as expected because any single compound cannot stand for the whole therapeutic efficacies of herbal medicines. Thus scientific studies should examine the effect of multiple constituents taken together rather than testing single compounds one at a time. It is believed that not all the components contribute to the efficacy of herbal medicines, and some constituents contribute largely to the effects. We assumed that the exact composition of combinatorial components accounting for the whole efficacy of original herbal medicines can be refined from the complex mixtures, and defined as bioactive equivalent combinatorial components (BECCs).

**Fig. 6:**
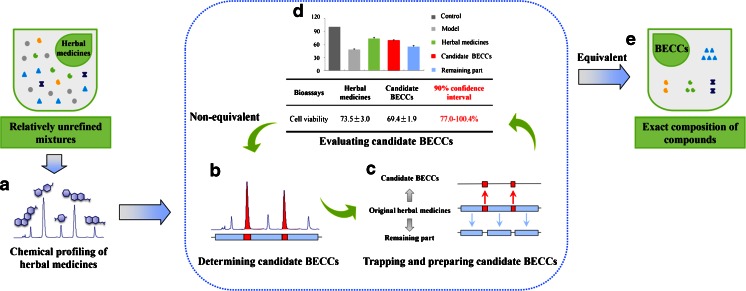
A schematic illustration of bioactive equivalence oriented feedback screening method. The operations include chemical profiling of herbal medicines, followed by an iterative loop of determining, collecting and evaluating candidate BECCs. (**a**) Herbal medicines of interest are chemically profiled. (**b**) Candidate BECCs are determined from original herbal medicines according to a predefined selection criterion. (**c**) Candidate BECCs are trapped and prepared, the remaining part is obtained at the same time. (**d**) Evaluate the bioactivity of candidate BECCs and assess bioactive equivalence between candidate BECCs and original herbal medicines. If not bioactive equivalent, candidate BECCs will be reselected until they achieve bioactive equivalence with original herbal medicines. (**e**) The BECCs are identified which can account for the whole therapeutic efficacy of original herbal medicines. *BECCs* bioactive equivalent combinatorial components.

To discover BECCs from herbal medicines of interest, we have established a feedback screening method, as illustrated in Fig. 6. The operations include chemical profiling of herbal medicines, followed by an iterative loop of determining, collecting and evaluating candidate BECCs.

To profile the chemical constituents in complex herbal medicines, diverse chromatographic methods can be used, such as high-performance liquid chromatography (HPLC) coupled with ultraviolet detection (UV), evaporative light scattering detector (ELSD) or mass spectrometry (MS). Furthermore, quadrupole time-of-flight mass spectrometry (Q-TOF MS) followed by database searching can provide structural information for unknown components.

The next step of the screening method is to determine which components in herbal medicines as temporally separated by liquid chromatography are candidates for inclusion in BECCs. In general, selection criteria for candidate BECCs are flexible. While normalized peak area ratio (%) was chosen as the selection criterion in this work, identified chemical structure, chemical polarity, or binding affinity to bioactive macromolecules can all be considered as reference parameters (34–36). Indeed, any components of interest or their combinations can be selected and evaluated.

To obtain a combination of candidate BECCs based on a predefined criterion, we engineered a real-time components trapping and combining system (Figure S1, Supplementary Material). Candidate BECCs and the remaining parts of herbal medicines can be prepared at the same time and used for bioactivity evaluation. Importantly, the concentration and proportion of constituents in candidate BECCs remained the same as that in original herbal medicines for bioactivity assay. Because the preparation process may potentially lead to sample loss, the investigation of the preparative recovery and repeatability for candidate BECCs is essential to ensure the feasibility and reliability of the preparation system.

To address the issue of comparing efficacy of the candidate BECCs with the original herbal medicines, we propose a concept of bioactive equivalence that is similar to bioequivalence in pharmacokinetics (37). Candidate BECCs are considered to be bioactive equivalents with original herbal medicines if the ratio of their efficacies falls within an acceptable range for a given assay. In this study, the efficacies of candidate BECCs and original CP were evaluated in three cell models and a rat model of MI. According to the efficacy assessment, selection criteria were stepwise adjusted until the candidate BECCs could achieve bioactive equivalence with original herbal medicines.

A combination of 18 compounds was identified as BECCs from CP using the bioactive equivalence oriented feedback screening method. We suggest that the developed screening method and the discovered BECCs can be applied for the selection of chemical markers for quality control of herbal medicines. Chemical markers are key to ensure the efficacy, safety and batch-to-batch consistency of herbal medicines. In most cases, due to insufficient chemical and pharmacological data for herbal medicines, lack of evidence-based chemical markers remains a major challenge for the quality control of herbal medicines (38,39). Using the described bioactive equivalence oriented feedback screening method, we can assess the rationality of designed chemical markers involved in Pharmacopeias and may suggest more suitable chemical markers. It is worth noting that the content of BECCs was 15.0% (w/w) of the original CP, and could be considered as the “defined labeled amount” of active constituents for CP against MI. It is promising that this screening method has a great potential in promoting standardization of herbal medicines. There are other possible applications of this method, for example in the assessment of toxic compounds or the analysis of integrative mechanisms of unrefined mixtures. The successful identification of naturally existing and integrative BECCs from the original efficacious formula leads us to suggest that our screening method could be used for multicomponent-based drug design (40,41).

## Conclusion

In conclusion, using the bioactive equivalence oriented feedback screening method, a combination of 18 compounds was identified as BECCs from Cardiotonic Pill (CP), which accounts for 15.0% (w/w) of original CP. We have demonstrated that the BECCs were as effective as CP in cell culture experiments and in a rat model of MI. Our future studies will focus on optimization of the BECCs and the elucidation of their multiple targets and possible synergistic mechanisms.

## Electronic supplementary material

Below is the link to the electronic supplementary material.

ESM 1 (PDF 0.98 MB)

## Abbreviations

BECCs: Bioactive equivalent combinatorial components
CCK-8: Cell counting kit-8
CK-MB: Creatine kinase-MB
CP: Cardiotonic Pill
HUVEC: Human umbilical vein endothelial cell
IL-6: Interleukin-6
LAD: Left anterior descending
LDH: Lactate dehydrogenase
LPS: Lipopolysaccharide
LV: Left ventricular
LVEDP: Left ventricular end diastolic pressure
LVSP: Left ventricular systolic pressure
MI: Myocardial infarction
MRCs: Mixture of reference compounds
Ox-LDL: Oxidized low density lipoprotein
PGE2: Prostaglandin E2
PLS-DA: Partial least square-discriminant analysis
ROS: Reactive oxygen species
SD: Standard deviation
SI: Simulated ischemia
TNF-α: Tumor necrosis factor-α
TTC: Triphenyltetrazolium chloride
