# Supplementary material for: Bioactive Equivalence of Combinatorial Components Identified in Screening of an Herbal Medicine
Source: Pharm Res. 2014 Feb 19;31(7):1788–800. doi: 10.1007/s11095-013-1283-1 (PMC4062815; doi:10.1007/s11095-013-1283-1)
Supplement: Supplementary file 1 — (PDF 0.98 MB) [file 11095_2013_1283_MOESM1_ESM.pdf]

Supplementary Material

Bioactive equivalence of combinatorial components identified in screening of an herbal medicine

Peng Liu · Hua Yang · Fang Long · Hai-Ping Hao · Xiaojun Xu · Ying Liu · Xiao-Wei Shi · Dan-Dan Zhang · Hao-Chuan Zheng · Qian-Ying Wen · Wen-Wen Li · Hui Ji · Xi-Juan Jiang · Bo-Li Zhang · Lian-Wen Qi · Ping Li

Peng Liu, Hua Yang and Fang Long contributed equally to this work.

P. Liu · H. Yang · F. Long · H.-P. Hao · X. Xu · Y. Liu · X.-W. Shi · D.-D. Zhang · H.-C. Zheng · Q.-Y. Wen · W.-W. Li · H. Ji · L.-W. Qi (*) · P. Li (*)

State Key Laboratory of Natural Medicines, China Pharmaceutical University, Nanjing 210009, China

e-mail: liping2004@126.com or fleude@126.com

X.-J. Jiang · B.-L. Zhang

Tianjin University of Traditional Chinese Medicine, Tianjin 300193, China

Bioactive equivalence evaluation by two one-sided *t*-test (1)**.** Let $\exp(\mu_B)/\exp(\mu_H) = \exp(\mu_B - \mu_H)$ be the ratio of the expected median values of the candidate BECCs and herbal medicines on the original scale. If the equivalence range is (0.7, 1.43), then the following test problem for equivalence is considered:

$$H_0 : \exp(\mu_B - \mu_H) \le 0.7 \quad \text{or} \quad \exp(\mu_B - \mu_H) \ge 1.43$$

$$H_1 : 0.7 < \exp(\mu_B - \mu_H) < 1.43$$

After logarithmic transformation, the above test problem becomes:

$$H_0 : \mu_B - \mu_H \le \ln 0.7 \quad \text{or} \quad \mu_B - \mu_H \ge \ln 1.43$$

$$H_1 : \ln 0.7 < \mu_B - \mu_H < \ln 1.43$$

Testing this two-sided equivalence problem is equivalent to simultaneous testing of the following two one-sided hypotheses (2):

$$H_{01} : \mu_B - \mu_H \le \ln 0.7 \quad \text{vs.} \quad H_{11} : \mu_B - \mu_H > \ln 0.7$$

and $H_{02} : \mu_B - \mu_H \ge \ln 1.43$ vs. $H_{12} : \mu_B - \mu_H < \ln 1.43$

Equivalence can be concluded at significance level 0.05, if both null hypotheses H_{01} and H_{02} are rejected at level 0.05, that is

$$T_{\ln 0.7} = \frac{\bar{Y}_B - \bar{Y}_H - \ln 0.7}{\hat{\sigma}_w \sqrt{\frac{1}{2}\left(\frac{1}{n_1} + \frac{1}{n_2}\right)}} > t_{0.95,\, n_1+n_2-2}$$

and $$T_{\ln 1.43} = \frac{\bar{Y}_B - \bar{Y}_H - \ln 1.43}{\hat{\sigma}_w \sqrt{\frac{1}{2}\left(\frac{1}{n_1} + \frac{1}{n_2}\right)}} < -t_{0.95,\, n_1+n_2-2}$$

where $t_{0.95,\, n_1+n_2-2}$ is the 0.95 quantile of the central *t*-distribution with $n_1 + n_2 - 2$ degrees of freedom, $\bar{Y}_B$ and $\bar{Y}_H$ are the least squares means of the candidate BECCs and original herbal medicines treatment and $\hat{\sigma}_W^2$ is the mean square, MS_{within}, from

the ANOVA after logarithmic transformation.

Rejection of H_{01} by $T_{\ln 0.7}$ at level 0.05 implies that

$$\frac{\overline{Y}_B - \overline{Y}_H - \ln 0.7}{\hat{\sigma}_W \sqrt{\frac{1}{2}\left(\frac{1}{n_1}+\frac{1}{n_2}\right)}} > t_{0.95,\, n_1+n_2-2}$$

In analogy, rejection of H_{02} by $T_{\ln 1.43}$ at level 0.05 implies that

$$\frac{\overline{Y}_B - \overline{Y}_H - \ln 1.43}{\hat{\sigma}_W \sqrt{\frac{1}{2}\left(\frac{1}{n_1}+\frac{1}{n_2}\right)}} < -t_{0.95,\, n_1+n_2-2}$$

Thus, rejection of H_{01} and H_{02} at level 0.05 is equivalent to:

$$\left[\overline{Y}_B - \overline{Y}_H - t_{0.95,\, n_1+n_2-2}\hat{\sigma}_W \sqrt{\frac{1}{2}\left(\frac{1}{n_1}+\frac{1}{n_2}\right)}, \overline{Y}_B - \overline{Y}_H + t_{0.95,\, n_1+n_2-2}\hat{\sigma}_W \sqrt{\frac{1}{2}\left(\frac{1}{n_1}+\frac{1}{n_2}\right)}\right] \subset (\ln 0.7, \ln 1.43)$$

Exponential transformation of the 90% confidence interval in the logarithmically transformed domain yields 90% confidence interval for the ratio of expected means $\exp(\mu_B - \mu_H)$ and the equivalent relationship:

$$\left[\exp\left(\overline{Y}_B - \overline{Y}_H - t_{0.95,\, n_1+n_2-2}\hat{\sigma}_W \sqrt{\frac{1}{2}\left(\frac{1}{n_1}+\frac{1}{n_2}\right)}\right), \exp\left(\overline{Y}_B - \overline{Y}_H + t_{0.95,\, n_1+n_2-2}\hat{\sigma}_W \sqrt{\frac{1}{2}\left(\frac{1}{n_1}+\frac{1}{n_2}\right)}\right)\right] \subset (0.7, 1.43)$$

Equivalence is concluded for the efficacies in a given bioassay at level 0.05, if the 90% confidence interval for $\exp(\mu_B - \mu_H)$ is included in the bioactive equivalence range.

References

(1) Hauschke D, Steinijans V, Pigeot I. In: Senn S, Scott M, Bloomfield P, Barnett V,

editors. Bioequivalence studies in drug development: methods and applications. Chichester: John Wiley & Sons; 2007. pp. 89-91.

(2) Schuirmann DJ. A comparison of the two one-sided tests procedure and the power approach for assessing the equivalence of average bioavailability. J Pharmacokinet Biopharm. 1987;15(6), 657-80.

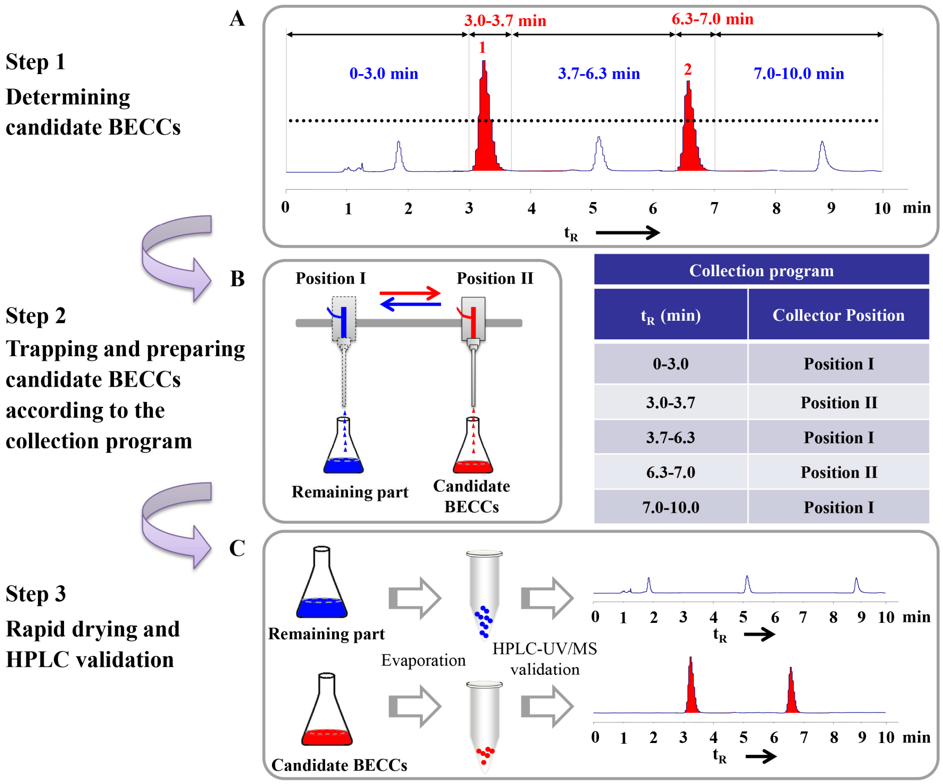

Figure S1 Preparation of candidate BECCs using real-time components trapping and combining system. (A) Candidate BECCs (depicted in red) were determined on the chromatogram according to the selection criterion. Retention time was recorded to form a collection program. (B) According to the collection program, the auto-collector moved to position II to prepare candidate BECCs while collecting remaining part (the collection of herbal medicines without candidate BECCs, depicted in blue) at position I. (C) The collected samples were dried quickly and subsequently validated by HPLC analysis. BECCs: bioactive equivalent combinatorial components.

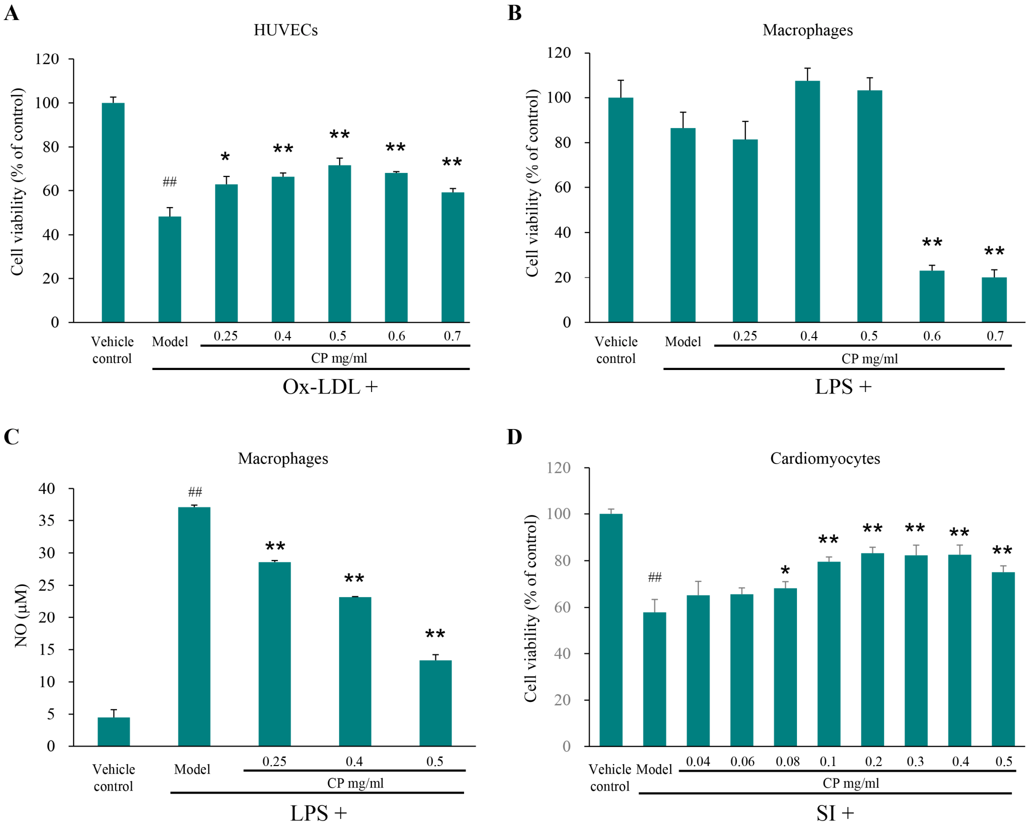

Figure S2 *In vitro* activity assays of different concentrations of Cardiotonic Pill. (A) Effects of different concentrations of Cardiotonic Pill on Ox-LDL-induced HUVEC injuries (Ox-LDL 70 μg/ml). The cell viability (as % of control) was assayed by CCK-8. (B) Cytotoxicity of different concentrations of Cardiotonic Pill on RAW 264.7 cells in the presence of LPS (1 μg/ml), measured by CCK-8. (C) Effects of different concentrations of Cardiotonic Pill on LPS-induced NO production in RAW 264.7 macrophages (LPS 1 μg/ml). NO production was determined by Griess reaction. (D) After 6h of simulated ischemia, cell viability of H9c2 cardiomyoblasts was assayed by CCK-8. Results are expressed as mean ± SD of at least three independent experiments. $*P < 0.05$, $**P < 0.01$ versus model group. ## $P < 0.01$ versus vehicle control group (one-way ANOVA, Dunnett test). Cells in model group were untreated. CP: Cardiotonic Pill; Ox-LDL: oxidized low density lipoprotein; LPS: lipopolysaccharide; SI: simulated ischemia.

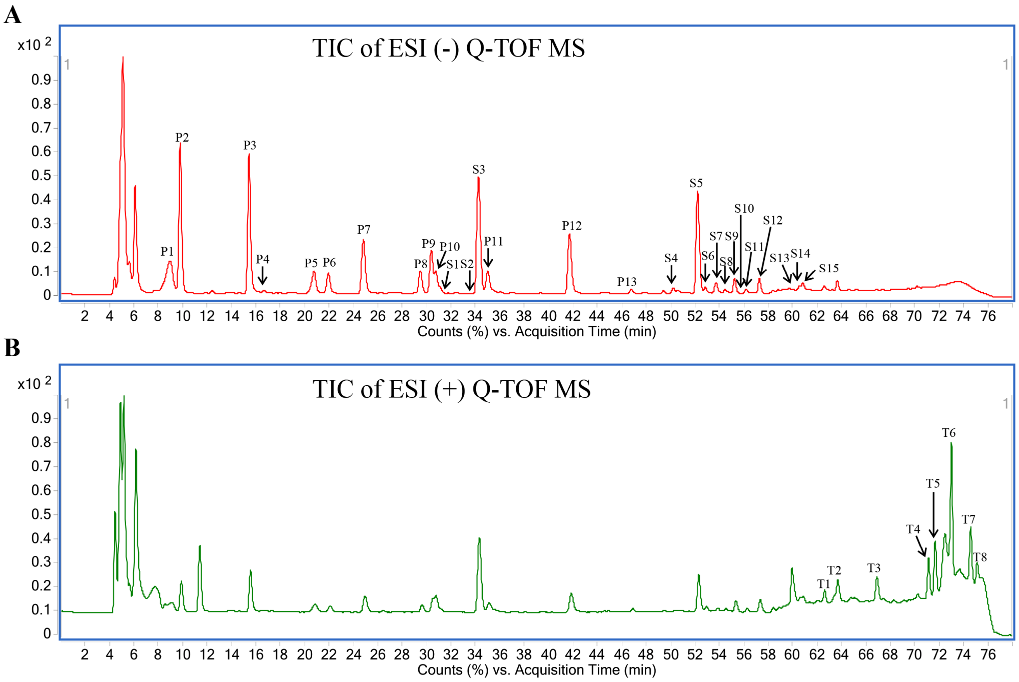

Figure S3 Total ion chromatograms of Cardiotonic Pill. Total ion chromatograms of Cardiotonic Pill by Q-TOF MS in (A) negative ion mode (B) positive ion mode. P1-P13: phenolic acids; S1-S15: saponins; T1-T8: tanshinones.

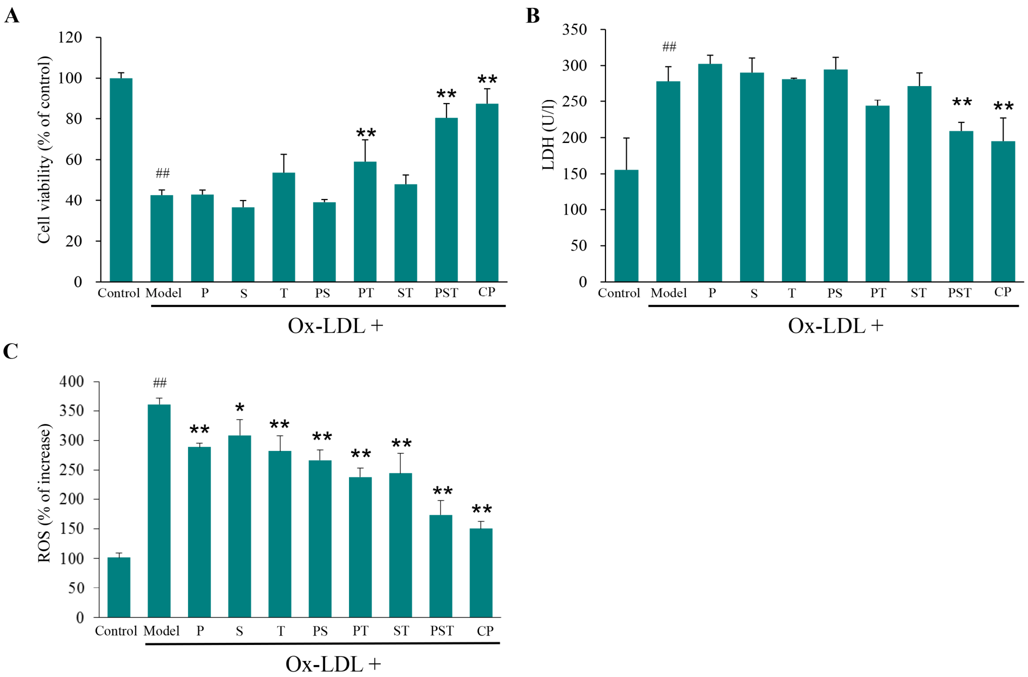

Figure S4 Protective effects of different groups of ingredients in BECCs on HUVECs against Ox-LDL injury. HUVEC injury was induced by pretreatment with Ox-LDL for 24 h and effects of different groups in BECCs on (A) cell viability, (B) LDH and (C) ROS were evaluated. Cell viability (as % of control) was assayed by CCK-8. Supernatant of HUVECs was collected for LDH assay by a commercial kit. ROS production (as % of increase) in HUVECs was determined by a fluorometric assay using DCFH-DA as a probe. Results are expressed as mean ± SD of at least three independent experiments. *$P < 0.05$, **$P < 0.01$ versus Ox-LDL group. ## $P < 0.01$ versus vehicle control group (one-way ANOVA, Dunnett test). Cells in model group were untreated. BECCs: bioactive equivalent combinatorial components. P: phenolic acids; S: saponins; T: tanshinones; PS: phenolic acids and saponins; PT: phenolic acids and tanshinones; ST: saponins and tanshinones; PST: phenolic acids, saponins and tanshinones (BECCs); CP: Cardiotonic Pill; Ox-LDL: oxidized low density lipoprotein.

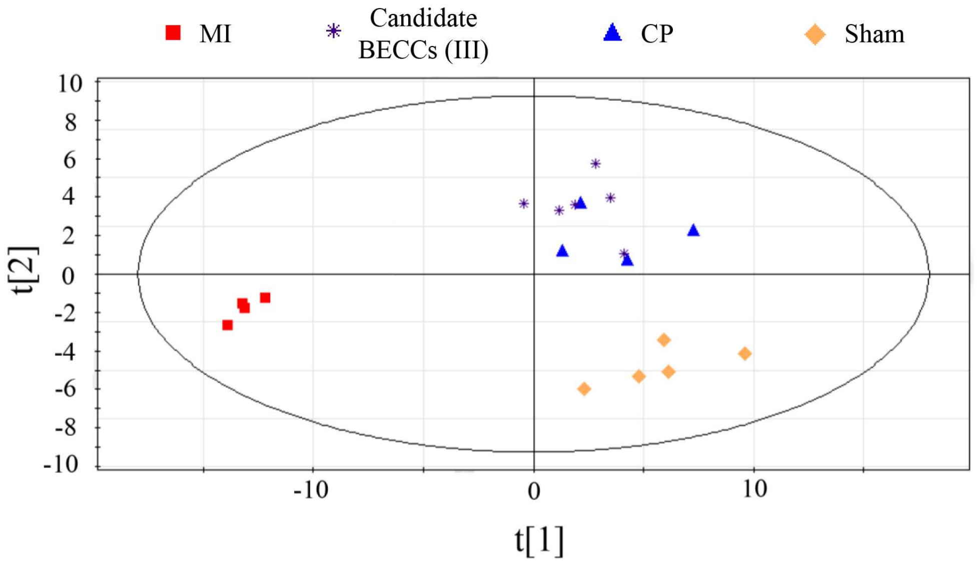

Figure S5 PLS-DA score plot for Cardiotonic Pill and candidate BECCs (III) treatments. The profiles of the metabolites in rat myocardial tissue within the infarct area were determined using gas chromatography/time-of-flight mass spectrometry (GC/TOF MS). MI: myocardial infarction; BECCs: bioactive equivalent combinatorial components; CP: Cardiotonic Pill; Sham: Sham operated.

Table S1 Preparative recovery and RSD of 18 compounds in candidate BECCs during the trapping and combining process

No.	Analytes	*Preparative Recovery (%)	RSD (%) (n = 5)
P2	Tanshinol	94.3	1.37
P3	Protocatechuic aldehyde	97.9	6.65
P5	Isolithospermic acid A	89.6	3.93
P6	Isolithospermic acid B	91.1	6.57
P7	Salvianolic acid D	87.7	4.62
P8	Salvianolic acid G	93.5	5.66
P9	Rosmarinic acid	96.3	4.18
P10	Lithospermic acid	91.9	1.88
S3	Ginsenoside-Rg1	94.5	6.15
P11	Salvianolic acid B	92.5	3.87
P12	Salvianolic acid A	88.2	2.94
S5	Ginsenoside-Rb1	92.2	0.25
S9	Ginsenoside-Rh1	96.4	4.36
S12	Ginsenoside-Rd	91.0	2.45
T3	Dihydrotanshinone I	95.7	4.59
T4	Tanshinone I	95.0	0.68
T5	Cryptotanshinone	90.7	4.10
T7	Tanshinone IIA	93.3	5.88
Average		92.9	3.89

*Preparative recovery = $C_B/C_C \times 100\%$, C_B is the content of compound in candidate BECCs, while C_C is the content of the same compound in Cardiotonic Pill. BECCs: bioactive equivalent combinatorial components.

Table S2 Calibration curve, r^2, test range, concentration and content of 18 compounds in candidate BECCs

No.	Analytes	Calibration curve	r^2	Test range (μg/ml)	Concentration (μg/mg) (*n* = 6)	Content (%) (*n* = 6)
P2	Tanshinol	y=95.5130x+101.6900	0.9999	6.31-151.41	26.71	2.67
P3	Protocatechuic aldehyde	y=61.4370x-11.3840	0.9997	9.84-315.00	10.39	1.04
P5	Isolithospermic acid A	y=20.6920x-53.4800	0.9999	7.66-245.00	11.49	1.15
P6	Isolithospermic acid B	y=26.4550x-6.3905	0.9999	6.09-195.00	7.90	0.79
P7	Salvianolic acid D	y=39.3480x-38.8370	0.9997	6.09-195.00	9.96	1.00
P8	Salvianolic acid G	y=26.2820x+26.2820	0.9998	5.03-161.00	7.00	0.70
P9	Rosmarinic acid	y=79.2080x+47.0880	0.9999	4.40-111.00	4.10	0.41
P10	Lithospermic acid	y=72.8320x+18.5140	0.9999	1.50-48.00	0.80	0.08
S3	Ginsenoside-Rg1	y=4.0791x-8.9264	0.9997	7.93-507.50	20.50	2.05
P11	Salvianolic acid B	y=73.5650x+49.9250	0.9998	4.53-145.00	5.09	0.51
P12	Salvianolic acid A	y=50.9130x+100.1800	0.9997	8.78-281.00	9.39	0.94
S5	Ginsenoside-Rb1	y=2.9303x-2.1224	0.9999	5.16-330.00	23.60	2.36
S9	Ginsenoside-Rh1	y=5.9709x-0.2279	0.9999	2.46-157.50	7.70	0.77
S12	Ginsenoside-Rd	y=3.6547x+2.2859	0.9997	2.11-135.00	2.43	0.24
T3	Dihydrotanshinone I	y=32.2160x-0.6432	0.9999	1.98-47.40	0.76	0.08
T4	Tanshinone I	y=42.7320x-1.6745	0.9999	1.15-27.0	1.02	0.10
T5	Cryptotanshinone	y=22.4840x+12.7970	0.9999	1.06-25.50	0.50	0.05
T7	Tanshinone IIA	y=37.3900x+2.3311	0.9999	0.95-22.80	0.81	0.08
Total						15.0

BECCs: bioactive equivalent combinatorial components.
